# Abortion—Large Quantity of Jelly

**Published:** 1874-01

**Authors:** A. J. Shaffer

**Affiliations:** Lawrenceville, Ga.


### CASE.

### EXPULSION SIX WEEKS FETUS, TOGETHER WITH LARGE QUANTITY OF JELLY.

BY A. J. SHAFFER, M.D., LAWRENCEVILLE, GA.

Mrs. M., æt. twenty-six, six years married, three children, all living and vigorous; last confined in September, 1871; enjoyed good health since. Her menses were regular and normal; appeared at usual time, 6th December, 1872, and without pain, but the flow continued up to the 3d January, 1873, when I was consulted by the husband, as to the persistent *metrostaxis*, which was greatly debilitating her. It was suggested that the flow might be symptomatic of commencing abortion, to which Mr. M. demurred, saying there was no evidence whatever of conception having taken place. He promised to return in a few days if there was not improvement.

18th January—Summoned in great haste, as Mrs. M. was 'wasting" very profusely. I found her quite weak and pallid—skin of waxy hue; the hemorrhage was very free. The parieties of the abdomen were distended to a degree usual at seven months of utero-gestation; flabby and soft. There was no pain.

On digital exploration, the uterus was found to be expanded into a globular ovoid, filling the superior strait of the pelvis; the cervix was quite deployed. The os presented a diameter of about three-eighths inch, was firm and unyielding, but by perseverance, finally relaxed so as to admit the index finger to the second joint, in which position nothing could be detected by the touch within the uterus. Upon withdrawing the finger a free hemorrhage followed, which excited some apprehensions for the safety of the patient.

I at once introduced the intra-uterine scoop, * which passed without obstruction to the distance of eight inches, and used it as a probe, sounding roughly the uterine cavity in every part. There appeared to be no foreign body within the uterus; there was no tenderness of the distended organ except in the region of attachment of the fallopian tube upon either side. I then threw open the bowl of the scoop and making careful search, found nothing adhering to the anterior or lateral walls of the uterus. Directing, now, the instrument to the posterior mucous wall, which seemed to have formed a distinct *cul-de-sac*, at the first essay, the scoop hung upon an apparently unyielding substance, but, after somewhat persistent traction, I closed the instrument and brought away a small portion of a rather opaque, jelly-like substance interspersed with strong fibrous threads, and slightly tinged with blood.

The scooping process was continued, progressively, detaching the material mentioned, while I retained the left index in proximity with the os, acting as a guide for the scoop. In ten or fifteen minutes active uterine contractions occurred, expelling large quantities of the gelatinous material. The scooping was kept up for three-fourths of an hour, when the instrument seemed to pass freely and smoothly over every part of the mucous lining of the uterus, and the uterine contractions no longer expelled anything. The instrument was now withdrawn, but before rising from the chair I made a careful examination and discovered a diminutive *foot* protruding from the os.

A six weeks fetus was expelled, and the uterus contracted well. The quantity of gelatinous material was not accurately weighed or measured, but was approximatively estimated at one gallon. The patient was greatly prostrated, and six months elapsed ere she regained robust health.

The question may be asked: Whence this strange freak of nature? The writer, entertaining some peculiar views in regard to the position of placental attachments in utero, will take occasion, at some future time, to lay these views before the profession.

---

* The instrument devised by me some years ago, and exhibited at the meeting of the State Medical Association at Columbus, in April, 1872.

